# Water-Resistant Casein-Based Adhesives for Veneer Bonding in Biodegradable Ski Cores

**DOI:** 10.3390/polym12081745

**Published:** 2020-08-05

**Authors:** Ronald Schwarzenbrunner, Marius Catalin Barbu, Alexander Petutschnigg, Eugenia Mariana Tudor

**Affiliations:** 1 Forest Products Technology and Timber Construction Department, Salzburg University of Applied Sciecnces, Markt 136a, 5431 Kuchl, Austria; rschwarzenbrunner.htw-m2019@fh-salzburg.ac.at (R.S.); marius.barbu@fh-salzburg.ac.at (M.C.B.); alexander.petutschnigg@fh-salzburg.ac.at (A.P.); 2 Transilvania University of Brasov, B-dul. Eroilor nr. 29, 500036 Brasov, Romania

**Keywords:** casein, veneer bonding, biodegradable gluing, ski core

## Abstract

The aim of this study is to investigate the performance of casein-based adhesives for the bonding of ash (*Fraxinus* spp.) veneers for the manufacture of biodegradable skis. Different formulations containing casein powder, water, lime, sodium silicate, and various glue amounts were tested for shear strength after water storage, modulus of rupture and modulus of elasticity, water absorption, and thickness swelling. Two other classic wood adhesives, namely epoxy and polyvinyl acetate (PVAc) type D4 were used as control. The highest efficiency of both mechanical and physical properties was recorded for the samples glued with caseins and an increased amount of lime. There was also an affinity between casein adhesive distribution and physical and mechanical plywood performance. Moreover, the developed casein-based glues were also used to bond the plywood for ski cores and tested in real-life winter conditions.

## 1. Introduction

The main intrinsic incentives associated to the enhancement of research on alternative adhesives for wood composites are the increased concern for bio-based glues and the health hazards resulting from the emissions from synthetic polymers [1,2].

One of the most common adhesives for wood-based materials is urea-formaldehyde (UF) [3]. It is used because of the high reactivity, colorlessness, rapid curing, and low cost [2,4]. UF is considered to be probably carcinogenic to humans [5,6,7,8].

The constant interest for formaldehyde free glues has been an important driver for the comeback of protein-based adhesives [9,10,11]. The overshadowed animal binders from collagen, blood, and casein have been used for centuries. The fish glues have been used since the 1800s and the soy glues since the 1900s [12]. Other natural adhesives are starch, tree gum, clays, cellulose, lignin, tannin, pitch, and dextrines [13,14,15,16].

Milk proteins are classified as caseins or whey proteins, depending on the different solubilities at a pH value of 4.6 [1], with notable adhesive properties. For thousands of years, milk proteins have been used as main ingredients in natural adhesive formulations [13,17,18]. They were substituted by synthetic polymers because of the cost [19], the easier processing [20], and the increased demands for milk protein for use in the food industry [1,13].

The use of casein as a glue for wood dates back to ancient Egypt [1] or the Middle Ages, as woodworking cement, when craftsmen employed it to bind together thin panels into thicker panels for paintings [21] or for the manufacture of Stradivari violins [22]. During the First World War, the plywood utilized in aircrafts was glued with casein adhesives [23,24,25] because of its good bond strength; nowadays, this protein adhesive is used for gluing bottle labels or cigarette papers [1,26] or is employed in high quality paper finishing, for antistatic coating of natural and synthetic fibers, and for indoor applications (plywood, door panels, laminates) [19]. Casein was considered a structural wood adhesive and was used also in the production of glulam beams [27].

Casein is procurable as powder [28]. To solubilize the casein, the formulation includes soaking in water and, after it has swollen, blending with an alkali [1]. The sodium silicate extends the pot life and the adhesion is increased by riveting tannate or alkali tannate [19]. The viscosity can be lowered by decreasing the hydrogen bonds, adding urea and ammonia [29].

The aim of this work was to analyze the suitability of casein glues as an alternative to conventional adhesives for the bonding of veneers as main raw material for biodegradable ski construction. The focus of the research was set on biodegradable and nontoxic ingredients.

## 2. Materials and Methods

**Figure 1:**
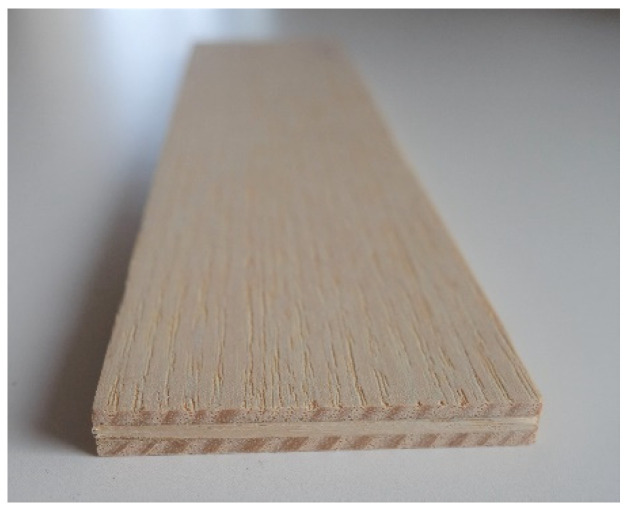
Sample of 3-layer plywood bonded with casein-based adhesive.

The ash veneers (*Fraxinus* spp.), 1.44 mm thick, with a moisture content of 8.42%, were supplied from the company J. u. A. Frischeis GmbH (Salzburg, Austria), to produce the 3-layer plywood for all samples (Figure 1). The veneer was glued with a casein recipe we developed on our own. The formulation for the glues consisted of casein powder (lactic-acid precipitated), water (pH value 7), and lime (soaked in water for 88 months, dry content: 60.9%), both from Kremer Pigmente (Aichstetten, Germany).

Two types of classical adhesives for wood gluing were used as reference: polyvinyl acetate (PVAc) D4 (Kleiberit 303) from Becker (Weingarten, Germany) and epoxy resin (Presto Epoxyharz) from Motip Dupli (Haßmersheim, Germany). Both are common because of their high resistance against water, their low cost (especially PVAc), high availability, and easy processing [28].

**Figure 2:**
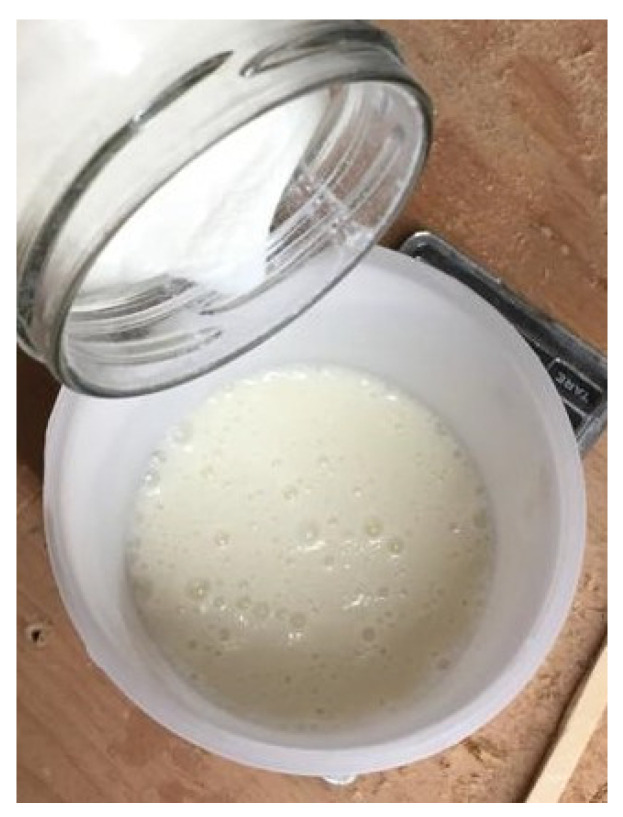
Preparing the adhesive based on casein powder, water, and lime.

The casein powder was soaked in water for four hours and subsequently mixed with lime (Figure 2). The ingredients were mixed manually at room temperature (20 °C and 35% relative air humidity) until it resulted in a uniform mass.

Previous research [28] has shown that the formulation based on “Casein 1” achieved the best bonding quality and subsequent appropriate mechanical properties (see Section 2.2. Testing Methods), compared to previous tested formulations. For this reason, “Casein 1” served as basis for the formulations studied in this research, with a glue amount of 600 g/m^2^.

The sample labeling was written as follows: the capital letter after “Casein 1” means a change in manufacturing process and the number corresponds to a higher water amount (“Casein 2”) and higher lime amount (“Casein 3”).

The casein powder of “Casein 1C” was just soaked for 1 h before mixing all ingredients. The glue was applied on veneer sheets directly after mixing, except for “Casein 1B”, utilized one hour later after blending (Table 1). 

For each type of plywood, the veneer was cut in 300 mm × 300 mm sheets, conditioned for one month at 20 °C and 35% relative air humidity. The higher percentage of air humidity of 65% was not appropriate, because of previous observations of Schwarzenbrunner (2019). The moisture content (m.c.) of the veneers was 8.42%. This level of m.c. should improve the penetration of the glue into the wood, to strengthen the bonding. When using casein glues, it is recommended to keep the m.c. of the veneers at about 8% [30].

### 2.1. Manufacturing of Plywood

Three-layered plywood boards were manufactured with 1.44 mm ash veneer, glued with the adhesives described in Table 2, and pressed with a Höfer HLOP 280 hydraulic laboratory press (Höfer Presstechnik, Taiskirchen, Austria). A total number of nine boards were produced.

After gluing the veneers with casein, four panels were pressed simultaneously with a pressing force of 342 kN for 16 h at 20 °C and 35% relative air humidity (Table 1 and Table 2). The glue application was 600 g/m^2^. Only “Casein 1D” had a 50% higher glue consumption and “Casein 1E” had a 50% lower glue consumption. This unusually high value resulted from the fact that the casein glue consists of more than 60% water, which slowly evaporates during hardening.

After another one-month conditioning at 20 °C and 35% relative air humidity, the finished panels were cut into specimens and then tested.

### 2.2. Testing Methods

Physical and mechanical properties of the samples glued with casein adhesive and reference resins were evaluated according to European norms. Prior to testing, the samples were conditioned at 20 °C and 65% relative air humidity until constant mass was reached.

The tests for mechanical properties were carried out using a Zwick Roell Z250 universal testing machine (ZwickRoell, Ulm, Germany), with following adjustment tensile of 0.973201 at a measurement range of 4000–20,000 N.

**Figure 3:**
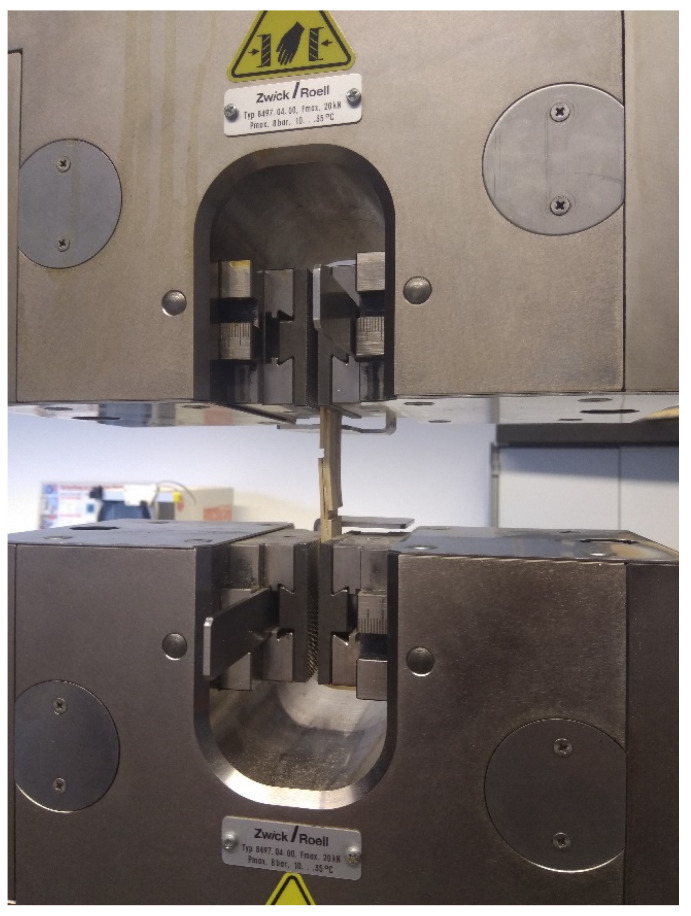
Shear strength test for 3-layer plywood with a Zwick Roell Z250 universal testing machine.

Ten specimens from each type of plywood board with 80 mm distance between clamps, 25 ± 0.5 mm shear width, 25 ± 0.5 mm shear length, and a saw cut width of 3 mm were cut for the tensile shear test (Figure 3) according to EN 314-1:2005-03 [31]. The samples were cut considering that the grain direction of the layer between the glue lines under test is perpendicular to the length of it. The pretreatment included water immersion of the samples (20 °C, pH value 7) for 24 h.

To determine the 3-point bending strength according to EN 310:2005-12-01 [32], six samples with 140, 50, and 4.5 mm length, width, and thickness were tested for each board.

**Figure 4:**
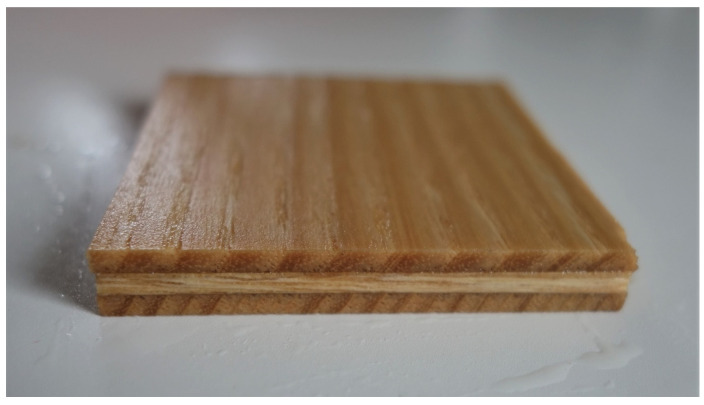
Sample with 3-layer plywood after 24 h water soaking.

Thickness swelling and water absorption were determined for eight samples with 50 mm by 50 mm per board (Figure 4). Their thicknesses and weight were measured at a level of accuracy of 0.1 mm and 0.01 g, according to EN 317:2005-12-01 [33]. In the next step, specimens were immersed for 24 h in water at 20 °C and pH level of 7. Subsequently, the samples were taken out and rinsed to eliminate excessive water. The samples were reweighed and the thickness measured again from the same location prior to water soaking.

## 3. Results

The samples bonded with epoxy resin achieved the highest tensile shear strength after water storage, which is not surprising, as this is a water-resistant thermoset (Table 3). “Casein 3” had the second highest tensile shear strength and a low dispersion after a 24-h water bath. This casein glue formulation with 16% lime achieved a 2.7 times higher tensile shear strength than the most waterproof PVAc glue (class D4). All shear strength values for “Casein 1” formulations were relatively similar, despite the various changes in manufacturing (“Casein 1C” soaked for 1 h in water before mixing with other ingredients of the formulation) and application (“Casein 1B” was utilized after one hour). The minimum value of shear strength for the “Casein 1” series was 0.18 MPa and the maximum value 0.86 MPa. “Casein 1D”, which had a higher glue amount (900 g/m^2^), achieved a 50% higher tensile shear strength than “Casein 1A”. For the samples with the same formulation and different application, “Casein 1A” and “Casein 1B”, a difference of 15% of the shear strength was measured. The “Casein 2” formulation with more water (74.53%) showed the weakest glue joint, with a minimum of 0.08 MPa and a maximum of 0.19 MPa.

The modulus of elasticity (MOE) of all tested specimens differed only slightly. If PVAc is ignored, all formulations varied less than 20% among themselves (Table 4). The most elastic formulations were PVAc (31.4% higher MOE than “Casein 1E”) followed by “Casein 3”, epoxy, “Casein 1C”, and “Casein 2”. The stiffest were all “Casein 1” formulations, except “Casein 1C”.

The flexural strength (MOR) of all tested specimens differed only slightly (less than 10% without considering “Casein 1D”) (Table 4). Following the same trend as for MOE, “Casein 1E”, the formulation with a higher glue amount, had the lowest MOR of just 61.5% (lower than that of the specimen with the highest value, which was “Casein 3”).

Analyzing the water absorption after 2 h water immersion (Table 5), the lowest mean was measured for the sample glued with epoxy resin: 13.15%, with a standard deviation of 1.1%. From all the samples manufactured with casein glue, the “Casein 1B” had the lowest mean value of 16.75%, with a standard deviation of 0.89% (10% lower compared with the values for the samples “Casein 1A”). The highest water absorption was reached for the samples “Casein 2”, which was 24.4% (0.98% standard deviation).

The thickness swelling after 2 h (Table 5) was the lowest for the “Casein 1D” samples, 4.39% (2.83% standard deviation), “Casein 2”, 4.88% (2.83% standard deviation), and “Casein 1A”, 5.65% (0.73% standard deviation). The reference samples bonded with epoxy resin and PVAc recorded higher means for thickness swelling, 15.48% and 9.96%, respectively.

In the case of water absorption after 24 h water storage, the samples glued with epoxy had the lowest values, with an average of 40.24% (Table 6). Of all casein glues, “Casein 1B” (left standing for 1 h after mixing) showed the lowest water uptake, with a minimum of 45.93%. Casein glues with a higher glue application (“Casein 1D”) or a higher lime amount (“Casein 3”) had just about 5% higher results. If the casein for the “Casein 1” formulation was only soaked for 1 h (“Casein 1C”), the water absorption was 9.5% higher compared to the case when it was soaked for 4 h (“Casein 1A”). The highest water absorption was measured for the samples with a lower glue application (“Casein 1E”) and with a higher water amount (“Casein 2”). Results of PVAc showed approximately the mean value of all results.

In the case of thickness swelling after 24 h water immersion (Table 6), with the exception of the “Casein 1E” samples (with less casein adhesive amount), all the other samples glued with casein recorded values at least 34% to 46% lower than that of epoxy and PVAc glued plywood.

## 4. Discussion

For all the tests, the differences between the minimum and maximum values are due to the cutting plan. For each test, the samples were taken from different positions of the 3-layered veneers. The differences might be caused also by the natural inhomogeneities of the wood; hence, the glue application may not be homogenous.

The bonding with epoxy resin offered the samples the highest tensile shear strength of 2.35 MPa. Adding more lime in the adhesive’s formulation (17%) conferred the samples “Casein 3” the highest values of the tensile shear strength, about two times bigger than the series “Casein 1”.

The modulus of elasticity was increased when PVAc was used to glue the three layers of veneer (9.45 N/mm²), but in the case of the epoxy adhesive, the results were similar to the samples glued with casein from series “Casein 3” and “Casein 1C” (about 105 N/mm²).

The trend for water absorption and thickness swelling after 2 and 24 h was similar. The lowest values were measured for samples “Casein 1B”, “Casein 1D”, and “Casein 3”, with no considerable differences. That means that the tested specimens soaked for 24 h absorbed about three times more water compared to the values measured after 2 h. For the samples with the same formulation and different application (”Casein 1A” and “Casein 1B”—left standing for one hour after mixing) a difference of 33% of the thickness swelling was measured.

One very important physical property of the casein-bonded 3-layer plywood is the thickness swelling. The samples “Casein 1D” and “Casein 2” had for both tests (after 2 and 24 h) a thickness swelling less than 5%. Moreover, for the series “Casein 1A” a level of 6% thickness swelling is still significantly low.

## 5. Conclusions

The casein glue mixtures are to be used effectively in the bonding of 3-layer ash plywood. The samples manufactured with formulations with a higher lime amount (over 10%) achieved the highest tensile shear strength after water storage. Their water-resistance is up to three times higher compared with the samples glued with PVAc class D4. Both values for modulus of rupture and modulus of elasticity were mostly higher for these testing specimens, compared with other samples bonded with other amounts of casein, water, and lime. Another important factor regarding mechanical and physical properties of the casein-bonded plywood is the amount of glue. An amount of 600 g/m^2^ glue showed the best performances.

Significant differences between the various formulations for casein-based glues were found, leading to the conclusion that there is an affinity between the ratio of the components, glue amount, and physical and mechanical plywood performance.

In summary, the samples bonded with “Casein 2” and “Casein 3” had the highest water resistance and mostly the highest mechanical properties of all casein glue formulations, also compared to PVAc (D4). Only the samples bonded with epoxy had a higher water resistance due to the duroplast characteristics.

The big differences of variance coefficients, namely for shear strength between 3.33% and 55.55%, MOE between 4.56% and 21.47%, MOR between 5.76% and 28.02%, and thickness swelling after 24 h between 12.81% and 81.97% are due to the inhomogeneous glue spread on the veneer surface, particularities of the ash wood, for example extractive content, pH value, wettability, with influence on the bonding strength, dimensional stability, and durability of adhesive joints [34].

We would also like to mention that the casein-bonded veneers were used for the sandwich construction of skis (core layer) and tested this year in the Austrian Alps, with carbon-fiber reinforcement. The testing of the skis is not the object of this study, but the gluability of casein glue on veneer sheets.

## Figures and Tables

**Table 1:** Adhesive formulations for the bonding of 3-layer ash plywood (4 mm thickness).

	Casein (%)	Water (%)	Lime (%)	Glue Amount (g/m^2^)
Epoxy	-	-	-	250
PVAc	-	-	-	150
Casein 1A	27.29	67.13	5.57	600
Casein 1B *	27.29	67.13	5.57	600
Casein 1C	27.29	67.13	5.57	600
Casein 1D	27.29	67.13	5.57	900
Casein 1E	27.29	67.13	5.57	300
Casein 2	20.51	75.30	4.19	600
Casein 3	24.01	59.07	16.92	600

* “Casein 1B” has the same formulation as “Casein 1A”, but was used one hour later after blending.

**Table 2:** Pressing parameters of reference glues.

	Pressing Time (min or h)	Pressing Temperature (°C)	Pressing Force (kN)
Epoxy	18 h	20	(clamps)
PVAc D4	15 min	50	135
All casein samples	16 h	20	342

**Table 3:** Mean, standard deviation, minimum, and maximum of the tensile shear strength of all test samples after water storage for 24 h, *n* = 10 (a, b, c, d values with the same letter are not significantly different: ANOVA, Post-Hoc Tukey HSD, α = 0.05); values are significantly different to the control (epoxy) (ANOVA; *p* < 0.05).

Tensile Shear Strength (MPa)				
Sample	Mean (Standard Deviation)	Minimum	Maximum	*p*
Casein 1A	0.51 ^a,b^ (0.21)	0.22	0.83	.
Casein 1B	0.60 ^a,b^ (0.24)	0.18	0.86	.
Casein 1C	0.49 ^a,b^ (0.16)	0.26	0.75	.
Casein 1D	0.76 ^b,c^ (0.06)	0.65	0.83	.
Casein 1E	0.16 ^a^ (0.04)	0.13	0.24	.
Casein 2	0.15 ^a^ (0.04)	0.08	0.19	.
Casein 3	1.20 ^c^ (0.04)	1.12	1.24	.
Epoxy	2.35 ^d^ (0.78)	1.24	3.68	-
PVAc D4	0.45 ^a,b^ (0.25)	0.16	0.76	.

**Table 4:** Mean, standard deviation, minimum, and maximum of the modulus of elasticity (MOE) and flexural strength (MOR) of all test samples, moisture content (m.c.) = 8.42%, *n* = 6 (a and b values with the same letter are not significantly different: ANOVA, Post-Hoc Tukey HSD, α = 0.05), values are significantly different to the control (epoxy) (ANOVA; *p* < 0.05).

Label	MOE (GPa)				MOR (MPa)			
	Mean (Standard Deviation)	Minimum	Maximum	*p*	Mean (Standard Deviation)	Minimum	Maximum	*p*
Casein 1A	7.60 ^a,b^ (1.12)	5.96	9.02	.	100.91 ^b^ (9.14)	89.25	114.78	-
Casein 1B	7.32 ^a^ (0.50)	6.74	8.19	.	101.73 ^b^ (5.40)	93.20	109.39	.
Casein 1C	8.41 ^a,b^ (1.18)	6.82	9.57	-	105.47 ^b^ (11.88)	86.48	121.95	.
Casein 1D	7.68 ^a,b^ (0.35)	7.07	8.03	-	96.17 ^b^ (5.54)	88.52	102.43	.
Casein 1E	7.19 ^a^ (0.89)	6.32	8.75	-	65.10 ^a^ (18.24)	40.81	96.32	.
Casein 2	8.10 ^a,b^ (0.74)	6.86	8.76	.	96.18 ^b^ (6.08)	87.96	105.43	-
Casein 3	8.57 ^a,b^ (1.84)	6.03	11.79	.	105.78 ^b^ (19.04)	75.11	134.62	.
Epoxy	8.50 ^a,b^ (1.06)	6.47	9.37	.	105.27 ^b^ (11.57)	82.56	114.86	-
PVAc D4	9.45 ^b^ (1.29)	8.00	11.22	.	99.25 ^b^ (20.58)	62.70	126.62	.

**Table 5:** Mean, standard deviation, minimum, and maximum of the water absorption and thickness swelling of all test samples after water storage for 2 h, *n* = 8 (a, b, c, d, e values with the same letter are not significantly different: ANOVA, Post-Hoc Tukey HSD, α = 0.05), values are significantly different to the control (epoxy) (ANOVA; *p* < 0.05).

	Water Absorption (%)				Thickness Swelling (%)			
	Mean (std. dev.)	Minimum	Maximum	*p*	Mean (std. dev.)	Minimum	Maximum	*p*
Casein 1A	18.56 ^b,c,d^ (1.10)	16.52	19.83	.	5.65 ^a^ (0.73)	4.32	6.47	.
Casein 1B	16.75 ^b^ (0.89)	15.30	17.97	.	8.52 ^a^ (1.75)	5.15	10.02	.
Casein 1C	20.46 ^d^ (1.80)	17.17	22.66	.	8.90 ^a^ (2.34)	4.96	12.13	.
Casein 1D	17.61 ^b,c^ (0.58)	16.65	18.46	.	4.39 ^a^ (2.83)	0.16	8.06	.
Casein 1E	22.89 ^e^ (1.98)	20.14	26.06	.	10.26 ^a,b^ (1.41)	8.77	12.61	-
Casein 2	24.40 ^e^ (0.98)	23.29	25.85	.	4.88 ^a^ (2.83)	0.96	9.19	.
Casein 3	17.56 ^b,c^ (0.29)	17.26	17.92	.	9.54 ^a,b^ (4.29)	2.87	16.42	-
Epoxy	13.15 ^a^ (1.10)	11.49	14.58	-	9.96 ^a,b^ (1.64)	8.09	12.86	-
PVAc D4	19.05 ^c,d^ (0.92)	17.99	20.14	.	15.48 ^b^ (6.96)	6.65	27.24	.

**Table 6:** Mean, standard deviation, minimum, and maximum of the water absorption and thickness swelling of all test samples after water storage for 24 h, *n* = 8 (a, b, c, d, e values with the same letter are not significantly different: ANOVA, Post-Hoc Tukey HSD, α = 0.05), values are significantly different to the control (epoxy) (ANOVA; *p* < 0.05).

	Water Absorption (%)				Thickness Swelling (%)			
	Mean (std. dev.)	Minimum	Maximum	*p*	Mean (std. dev.)	Minimum	Maximum	*p*
Casein 1A	56.74 ^b,c,d^ (3.29)	50.68	60.62	.	6.01 ^a^ (0.77)	4.59	6.84	.
Casein 1B	50.71 ^b^ (2.70)	45.93	54.14	.	9.06 ^a^ (1.84)	5.53	10.68	.
Casein 1C	62.15 ^d^ (5.62)	51.89	68.94	.	9.49 ^a^ (2.49)	5.33	13.00	.
Casein 1D	53.18 ^b,c^ (1.60)	50.67	55.64	.	4.66 ^a^ (3.03)	0.21	8.67	.
Casein 1E	69.86 ^e^ (6.20)	61.13	79.72	.	10.93 ^a,b^ (1.49)	9.37	13.38	-
Casein 2	74.00 ^e^ (3.10)	70.25	78.48	.	4.77 ^a^ (3.91)	−1.72	9.91	.
Casein 3	53.50 ^b,c^ (0.85)	52.43	54.93	.	10.16 ^a,b^ (4.57)	2.98	17.43	-
Epoxy	40.24 ^a^ (3.33)	35.06	44.77	-	10.54 ^a,b^ (1.75)	8.52	13.63	-
PVAc D4	58.15 ^c,d^ (2.77)	54.74	61.45	.	16.46 ^b^ (7.41)	7.03	28.94	.
